# Supplementary material for: Educational inequalities in mortality amenable to healthcare. A comparison of European healthcare systems
Source: PLoS One. 2020 Jul 2;15(7):e0234135. doi: 10.1371/journal.pone.0234135 (PMC7332057; doi:10.1371/journal.pone.0234135)
Supplement: S1 Table — (DOCX) [file pone.0234135.s001.docx]

**Table S1: Data sources**

| **Country** | **Age group** | **Data** | **Period** | **Contact information** |
| --- | --- | --- | --- | --- |
| **Austria** | 35-79 | Longitudinal | 2001-2002 | Data were obtained from Statistics Austria ([info@statistik.gv.at](mailto:info@statistik.gv.at)). Our contact person in the project was  Johannes Klotz ([johannes.klotz@statistik.gv.at](mailto:johannes.klotz@statistik.gv.at)) |
| **Belgium** | 35-79 | Longitudinal | 2004-2005 | Data were obtained from Statistics Belgium ([statbel@economie.fgov.be](mailto:statbel@economie.fgov.be)). Our contact person in the project was Patrick Deboosere ([patrick.deboosere@vub.ac.be](mailto:patrick.deboosere@vub.ac.be)) |
| **Czech Republic** | 35-79 | CS, unlinked | 1998-2003 | Data were obtained from the Czech Statistical Office ([infoservis@czso.cz](mailto:infoservis@czso.cz)). Our contact person in the project was Jitka Rychtarikova ([rychta@natur.cuni.cz](mailto:rychta@natur.cuni.cz)) |
| **Denmark** | 35-79 | Longitudinal | 2001-2005 | Data were obtained through Statistics Denmark ([www.dst.dk](http://www.dst.dk)). Our contact person in the project was Anita Lange ([anl@dst.dk](mailto:anl@dst.dk)) |
| **England/ Wales** | 35-79 | Longitudinal | 2001-2006 | Data were obtained from the Office for National Statistics ([www.ons.gov.uk](http://www.ons.gov.uk)). Our contact person in the project was Chris White ([chris.white@ons.gov.uk](mailto:chris.white@ons.gov.uk)). |
| **Estonia** | 35-79 | CS, unlinked | 1998-2002 | Death data were obtained from Causes of Death Registry, National Institute for Health Development (NIHD), Tallinn, Estonia ([gleb.denissov@tai.ee](mailto:gleb.denissov@tai.ee)); population data by educational level were obtained from Statistics Estonia open database (<https://stat.ee>). Data were combined into multidimensional frequency tables for project needs by Mall Leinsalu, Department of Epidemiology and Biostatistics, NIHD ([mall.leinsalu@tai.ee](mailto:mall.leinsalu@tai.ee)). She is the contact person for further requests. |
| **Finland** | 35-79 | Longitudinal | 2000-2005 | Data were obtained from Statistics Finland (web: <http://www.stat.fi/tup/mikroaineistot/index_en.html>; [tutkijapalvelut@stat.fi](mailto:tutkijapalvelut@stat.fi)). Our contact person in the project was Pekka Martikainen ([pekka.martikainen@helsinki.fi](mailto:pekka.martikainen@helsinki.fi)) |
| **France** | 35-79 | Longitudinal | 1999-2004 | Death data were obtained from the French Causes of Death Registry, CepiDc, French National Institute for Health and Medical Research (Inserm; <https://www.inserm.fr>); population data by educational level were obtained from the French National Institute for Statistics (INSEE; <https://www.insee.fr/en/information/3974508>). Data were combined into multidimensional frequency tables for project needs by Gwenn Menvielle, Pierre Louis Institute for Epidemiology and Public Health ([gwenn.menvielle@inserm.fr](mailto:gwenn.menvielle@inserm.fr)). She is the contact person for further requests. |
| **Hungary** | 35-79 | CS, unlinked | 1999-2002 | Data were obtained from the Hungarian Central Statistical Office (<https://kapcsolat.ksh.hu/ContactCenter/index.xhtml?lang=en>). Our contact person in the project was Katalin Kovacs ([kovacs@demografia.hu](mailto:kovacs@demografia.hu)) |
| **Italy (Turin)** | 35-79 | Longitudinal | 2001-2006 | Data were obtained from the Turin Longitudinal Mortality Study. Our contact person in the project was Giuseppe Costa ([giuseppe.costa@epi.piemonte.it](mailto:giuseppe.costa@epi.piemonte.it)) |
| **Lithuania** | 35-69 | Longitudinal | 2001-2005 | Data were obtained from the Lithuanian Department of Statistics ([aleksandra.golubovic@stat.gov.lt](mailto:aleksandra.golubovic@stat.gov.lt)). Our contact persons in the project was Domantas Jasilionis ([Jasilionis@demogr.mpg.de](mailto:Jasilionis@demogr.mpg.de)) and Ramunė Kalėdienė ([Ramune.Kalediene@lsmuni.lt](mailto:Ramune.Kalediene@lsmuni.lt)) |
| **Norway** | 40-79 | Longitudinal | 2001-2006 | Norwegian Institute of Public Health, Kåre Bævre ( [kare.baevre@fhi.no](mailto:kare.baevre@fhi.no)). Our contact person in the project was Bjørn Heine Strand ([bjorn.heine.strand@fhi.no](mailto:bjorn.heine.strand@fhi.no)) |
| **Poland** | 35-64 | CS, unlinked | 2001-2003 | Data can be obtained from Statistics Poland (<https://stat.gov.pl/en/contacts/>. Our contact person in the project was Bogdan Wojtyniak ([bogdan@medstat.waw.pl](mailto:bogdan@medstat.waw.pl)) |
| **Scotland** | 35-74 | Longitudinal | 2001-2005 | Professor Chris Dibben ([chris.dibben@ed.ac.uk](mailto:chris.dibben@ed.ac.uk)), Longitudinal Study Centre Scotland, National Records of Scotland, Ladywell House, Ladywell Road Edinburgh Scotland EH12 7TF. |
| **Slovenia** | 35-79 | Longitudinal | 2002-2006 | Data can be obtained from the Statistical Office of the Republic of Slovenia ([gp.surs@gov.si](mailto:gp.surs@gov.si)). Our contact person in the project was Barbara Artnik ([barbara.artnik@mf.uni-lj.si](mailto:barbara.artnik@mf.uni-lj.si)) |
| **Spain (Barc.)** | 35-79 | CS, repeated | 2002-2006 | Spanish data for Barcelona city was obtained through Carme Borrell ([cborrell@aspb.cat](mailto:cborrell@aspb.cat)) |
| **Spain (Basque)** | 35-79 | Longitudinal | 2001-2006 | Regional Spanish data for the Basque country was obtained through Santiago Esnaola ([sesnaola@ej-gv.es](mailto:sesnaola@ej-gv.es)) |
| **Spain (Madrid)** | 35-79 | Longitudinal | 2001-2003 | Regional Spanish data for Madrid was obtained through Enrique Regidor ([eregidor@msc.es](mailto:eregidor@msc.es)) |
| **Sweden** | 35-79 | Longitudinal | 2000-2004 | Data can be obtained from Statistics Sweden (<https://www.scb.se/en/About-us/contact-us/>. Our contact in the project was Olle Lundberg ([olle.lundberg@chess.su.se](mailto:olle.lundberg@chess.su.se)) |
| **Switzerland** | 35-79 | Longitudinal | 2000-2005 | Swiss Federal Statistical Office, Espace de l'Europe 10, CH-2000 Neuchâtel. Demetriq project contact person: Matthias Bopp ([matthias.bopp@uzh.ch](mailto:matthias.bopp@uzh.ch)). Current contact person: Dominik Ullmann ([dominik.ullmann@bfs.admin.ch](mailto:dominik.ullmann@bfs.admin.ch)) |
