# Supplementary material for: Educational inequalities in mortality amenable to healthcare. A comparison of European healthcare systems
Source: PLoS One. 2020 Jul 2;15(7):e0234135. doi: 10.1371/journal.pone.0234135 (PMC7332057; doi:10.1371/journal.pone.0234135)
Supplement: S2 Table — (DOCX) [file pone.0234135.s002.docx]

**Table S2: Educational distribution**

| **Country** | **ISCED**  **0-2** | **ISCED**  **3-4** | **ISCED**  **5-6** |
| --- | --- | --- | --- |
| **Austria** | 30.8 | 56.05 | 13.2 |
| **Belgium** | 48.6 | 25.2 | 26.2 |
| **Czech Republic** | 59.5 | 29.1 | 11.4 |
| **Denmark** | 38.6 | 37.7 | 23.7 |
| **England/ Wales** | 38.9 | 25.8 | 24.0 |
| **Estonia** | 26.2 | 55.7 | 18.1 |
| **Finland** | 33.9 | 36.8 | 29.3 |
| **France** | 42.4 | 40.4 | 17.2 |
| **Hungary** | 43.6 | 43.2 | 13.2 |
| **Italy (Turin)** | 59.2 | 27.4 | 13.4 |
| **Lithuania** | 22.5 | 59.5 | 18 |
| **Norway** | 19.7 | 55.0 | 25.3 |
| **Poland** | 53.2 | 34.5 | 12.3 |
| **Scotland** | 44.7 | 31.0 | 24.3 |
| **Slovenia** | 45.0 | 31.5 | 23.5 |
| **Spain (Barc.)** | 55.7 | 21.5 | 22.8 |
| **Spain (Basque)** | 60.9 | 20.5 | 18.6 |
| **Spain (Madrid)** | 56.6 | 21.9 | 21.5 |
| **Sweden** | 27.1 | 50.3 | 22.6 |
| **Switzerland** | 19.8 | 57.8 | 22.4 |
