# Supplementary material for: Educational inequalities in mortality amenable to healthcare. A comparison of European healthcare systems
Source: PLoS One. 2020 Jul 2;15(7):e0234135. doi: 10.1371/journal.pone.0234135 (PMC7332057; doi:10.1371/journal.pone.0234135)
Supplement: S3 Table — (DOCX) [file pone.0234135.s003.docx]

**Table S3: Analysis of variance, RII and SII estimates of healthcare system types (amenable mortality)**

| **RII men** |  |  |  |  |  |  | **SII men** |  |  |  |  |  |  |
| --- | --- | --- | --- | --- | --- | --- | --- | --- | --- | --- | --- | --- | --- |
|  |  |  |  |  |  |  |  |  |  |  |  |  |  |
| *Groups* | *Count* | *Sum* | *Average* | *Variance* |  |  | *Groups* | *Count* | *Sum* | *Average* | *Variance* |  |  |
| HCS Type 1 | 6 | 16.6 | 2.77 | 0.97 |  |  | HCS Type 1 | 6 | 1437.5 | 239.6 | 21813.6 |  |  |
| HCS Type 2 | 3 | 6.38 | 2.13 | 0.03 |  |  | HCS Type 2 | 3 | 474.9 | 158.3 | 253.6 |  |  |
| HCS Type 3 | 3 | 6.27 | 2.09 | 0.24 |  |  | HCS Type 3 | 3 | 405.8 | 135.3 | 728.3 |  |  |
| HCS Type 4 | 4 | 14.3 | 3.57 | 1.42 |  |  | HCS Type 4 | 4 | 1657.9 | 414.5 | 38088.4 |  |  |
| *Source of Variation* | *SS* | *df* | *MS* | *F* | *P-value* | *F crit* | *Source of Variation* | *SS* | *df* | *MS* | *F* | *P-value* | *F crit* |
| Between Groups | 5.15 | 3 | 1.72 | 2.14 | 0.15 | 3.49 | Between Groups | 173555.2 | 3 | 57851.7 | 3.1 | 0.1 | 3.5 |
| Within Groups | 9.64 | 12 | 0.80 |  |  |  | Within Groups | 225297.2 | 12 | 18774.8 |  |  |  |
|  |  |  |  |  |  |  |  |  |  |  |  |  |  |
| Total | 14.78 | 15 |  |  |  |  | Total | 398852.4 | 15 |  |  |  |  |
| **RII women** |  |  |  |  |  |  | **SII women** |  |  |  |  |  |  |
|  |  |  |  |  |  |  |  |  |  |  |  |  |  |
| *Groups* | *Count* | *Sum* | *Average* | *Variance* |  |  | *Groups* | *Count* | *Sum* | *Average* | *Variance* |  |  |
| HCS Type 1 | 6 | 14.8 | 2.46 | 0.17 |  |  | HCS Type 1 | 6 | 749.3 | 124.9 | 3097.2 |  |  |
| HCS Type 2 | 3 | 6.56 | 2.19 | 0.09 |  |  | HCS Type 2 | 3 | 374.9 | 125.0 | 1201.2 |  |  |
| HCS Type 3 | 3 | 6.97 | 2.32 | 0.04 |  |  | HCS Type 3 | 3 | 284.2 | 94.7 | 106.1 |  |  |
| HCS Type 4 | 4 | 12.7 | 3.18 | 0.45 |  |  | HCS Type 4 | 4 | 837.4 | 209.4 | 10616.7 |  |  |
| *Source of Variation* | *SS* | *df* | *MS* | *F* | *P-value* | *F crit* | *Source of Variation* | *SS* | *df* | *MS* | *F* | *P-value* | *F crit* |
| Between Groups | 2.17 | 3 | 0.72 | 3.56 | 0.05 | 3.49 | Between Groups | 27441.5 | 3 | 9147.2 | 2.2 | 0.1 | 3.5 |
| Within Groups | 2.44 | 12 | 0.20 |  |  |  | Within Groups | 49950.5 | 12 | 4162.5 |  |  |  |
|  |  |  |  |  |  |  |  |  |  |  |  |  |  |
| Total | 4.61 | 15 |  |  |  |  | Total | 77392.0 | 15 |  |  |  |  |
