# Supplementary material for: Educational inequalities in mortality amenable to healthcare. A comparison of European healthcare systems
Source: PLoS One. 2020 Jul 2;15(7):e0234135. doi: 10.1371/journal.pone.0234135 (PMC7332057; doi:10.1371/journal.pone.0234135)
Supplement: S4 Table — (DOCX) [file pone.0234135.s004.docx]

**Table S4: Analysis of variance, RII and SII estimates of healthcare system types – excluding Switzerland and Lithuania (amenable mortality)**

| **RII men** |  |  |  |  |  |  | **SII men** |  |  |  |  |  |  |
| --- | --- | --- | --- | --- | --- | --- | --- | --- | --- | --- | --- | --- | --- |
|  |  |  |  |  |  |  |  |  |  |  |  |  |  |
| *Groups* | *Count* | *Sum* | *Average* | *Variance* |  |  | *Groups* | *Count* | *Sum* | *Average* | *Variance* |  |  |
| HCS Type 1 | 5 | 13.9 | 2.78 | 1.21 |  |  | HCS Type 1 | 5 | 1320.9 | 264.2 | 22725.0 |  |  |
| HCS Type 2 | 3 | 6.38 | 2.13 | 0.03 |  |  | HCS Type 2 | 3 | 474.9 | 158.3 | 253.6 |  |  |
| HCS Type 3 | 3 | 6.27 | 2.09 | 0.24 |  |  | HCS Type 3 | 3 | 405.8 | 135.3 | 728.3 |  |  |
| HCS Type 4 | 3 | 11.4 | 3.82 | 1.78 |  |  | HCS Type 4 | 3 | 1364.3 | 454.8 | 47393.9 |  |  |
| *Source of Variation* | *SS* | *df* | *MS* | *F* | *P-value* | *F crit* | *Source of Variation* | *SS* | *df* | *MS* | *F* | *P-value* | *F crit* |
| Between Groups | 5.85 | 3 | 1.95 | 2.19 | 0.15 | 3.71 | Between Groups | 191210.8 | 3 | 63736.9 | 3.4 | 0.1 | 3.71 |
| Within Groups | 8.91 | 10 | 0.89 |  |  |  | Within Groups | 187651.8 | 10 | 18765.2 |  |  |  |
|  |  |  |  |  |  |  |  |  |  |  |  |  |  |
| Total | 14.77 | 13 |  |  |  |  | Total | 378862.7 | 13 |  |  |  |  |
| **RII women** |  |  |  |  |  |  | **SII women** |  |  |  |  |  |  |
|  |  |  |  |  |  |  |  |  |  |  |  |  |  |
| *Groups* | *Count* | *Sum* | *Average* | *Variance* |  |  | *Groups* | *Count* | *Sum* | *Average* | *Variance* |  |  |
| HCS Type 1 | 5 | 12.61 | 2.52 | 0.18 |  |  | HCS Type 1 | 5 | 694.0 | 138.8 | 2415.8 |  |  |
| HCS Type 2 | 3 | 6.56 | 2.19 | 0.09 |  |  | HCS Type 2 | 3 | 374.9 | 125.0 | 1201.2 |  |  |
| HCS Type 3 | 3 | 6.97 | 2.32 | 0.04 |  |  | HCS Type 3 | 3 | 284.2 | 94.7 | 106.1 |  |  |
| HCS Type 4 | 3 | 9.54 | 3.18 | 0.68 |  |  | HCS Type 4 | 3 | 686.6 | 228.9 | 13643.6 |  |  |
| *Source of Variation* | *SS* | *df* | *MS* | *F* | *P-value* | *F crit* | *Source of Variation* | *SS* | *df* | *MS* | *F* | *P-value* | *F crit* |
| Between Groups | 1.74 | 3 | 0.58 | 2.48 | 0.12 | 3.71 | Between Groups | 30069.4 | 3 | 10023.1 | 2.5 | 0.1 | 3.71 |
| Within Groups | 2.34 | 10 | 0.23 |  |  |  | Within Groups | 39565.1 | 10 | 3956.5 |  |  |  |
|  |  |  |  |  |  |  |  |  |  |  |  |  |  |
| Total | 4.08 | 13 |  |  |  |  | Total | 69634.5 | 13 |  |  |  |  |
