# Supplementary material for: Educational inequalities in mortality amenable to healthcare. A comparison of European healthcare systems
Source: PLoS One. 2020 Jul 2;15(7):e0234135. doi: 10.1371/journal.pone.0234135 (PMC7332057; doi:10.1371/journal.pone.0234135)
Supplement: S5 Table — (DOCX) [file pone.0234135.s005.docx]

**Table S5: Analysis of variance, RII and SII estimates of healthcare system types (all-cause mortality)**

| **RII men** |  |  |  |  |  |  | **SII men** |  |  |  |  |  |  |
| --- | --- | --- | --- | --- | --- | --- | --- | --- | --- | --- | --- | --- | --- |
|  |  |  |  |  |  |  |  |  |  |  |  |  |  |
| *Groups* | *Count* | *Sum* | *Average* | *Variance* |  |  | *Groups* | *Count* | *Sum* | *Average* | *Variance* |  |  |
| HCS Type 1 | 6 | 16.5 | 2.75 | 0.95 |  |  | HCS Type 1 | 6 | 6344.2 | 1057.4 | 302874.7 |  |  |
| HCS Type 2 | 3 | 7.10 | 2.37 | 0.06 |  |  | HCS Type 2 | 3 | 2922.9 | 974.3 | 71229.6 |  |  |
| HCS Type 3 | 3 | 7.07 | 2.36 | 0.08 |  |  | HCS Type 3 | 3 | 2447.8 | 815.9 | 37061.2 |  |  |
| HCS Type 4 | 4 | 15.2 | 3.79 | 1.85 |  |  | HCS Type 4 | 4 | 7947.6 | 1986.9 | 319683.0 |  |  |
| *Source of Variation* | *SS* | *Df* | *MS* | *F* | *P-value* | *F crit* | *Source of Variation* | *SS* | *df* | *MS* | *F* | *P-value* | *F crit* |
| Between Groups | 5.04 | 3 | 1.68 | 1.90 | 0.18 | 3.49 | Between Groups | 3180950.0 | 3 | 1060316.7 | 4.73 | 0.02 | 3.49 |
| Within Groups | 10.6 | 12 | 0.88 |  |  |  | Within Groups | 2690004.3 | 12 | 224167.0 |  |  |  |
|  |  |  |  |  |  |  |  |  |  |  |  |  |  |
| Total | 15.6 | 15 |  |  |  |  | Total | 5870954.4 | 15 |  |  |  |  |
| **RII women** |  |  |  |  |  |  | **SII women** |  |  |  |  |  |  |
|  |  |  |  |  |  |  |  |  |  |  |  |  |  |
| *Groups* | *Count* | *Sum* | *Average* | *Variance* |  |  | *Groups* | *Count* | *Sum* | *Average* | *Variance* |  |  |
| HCS Type 1 | 6 | 11.6 | 1.93 | 0.06 |  |  | HCS Type 1 | 6 | 2209.1 | 368.2 | 24910.0 |  |  |
| HCS Type 2 | 3 | 6.87 | 2.29 | 0.01 |  |  | HCS Type 2 | 3 | 1820.1 | 606.7 | 14492.0 |  |  |
| HCS Type 3 | 3 | 6.78 | 2.26 | 0.04 |  |  | HCS Type 3 | 3 | 1288.6 | 429.5 | 6226.4 |  |  |
| HCS Type 4 | 4 | 10.4 | 2.60 | 0.13 |  |  | HCS Type 4 | 4 | 2553.5 | 638.4 | 59741.0 |  |  |
| *Source of Variation* | *SS* | *df* | *MS* | *F* | *P-value* | *F crit* | *Source of Variation* | *SS* | *df* | *MS* | *F* | *P-value* | *F crit* |
| Between Groups | 1.09 | 3 | 0.36 | 5.38 | 0.01 | 3.49 | Between Groups | 228870.4 | 3 | 76290.1 | 2.65 | 0.10 | 3.49 |
| Within Groups | 0.81 | 12 | 0.07 |  |  |  | Within Groups | 345209.9 | 12 | 28767.5 |  |  |  |
|  |  |  |  |  |  |  |  |  |  |  |  |  |  |
| Total | 1.90 | 15 |  |  |  |  | Total | 574080.3 | 15 |  |  |  |  |
