# Supplementary material for: Educational inequalities in mortality amenable to healthcare. A comparison of European healthcare systems
Source: PLoS One. 2020 Jul 2;15(7):e0234135. doi: 10.1371/journal.pone.0234135 (PMC7332057; doi:10.1371/journal.pone.0234135)
Supplement: S6 Table — (DOCX) [file pone.0234135.s006.docx]

**Table S6: RII and SII estimates in total (all-cause) mortality**

|  | Men | | Women | |
| --- | --- | --- | --- | --- |
|  | RII | SII | RII | SII |
| Austria | 1.91  (0.05) | 608.7  (25.5) | 1.54  (0.06) | 236.3  (17.0) |
| Belgium | 2.28  (0.04) | 845.0  (17.4) | 1.84  (0.05) | 357.6  (13.7) |
| Czech Republic | 4.66  (0.05) | 2110.0  (10.7) | 2.27  (0.03) | 639.9  (10.3) |
| Denmark | 2.10  (0.03) | 788.4  (14.9) | 2.08  (0.04) | 525.4  (12.4) |
| England/ Wales | 2.41  (0.14) | 734.9  (47.1) | 2.09  (0.17) | 447.9  (39.0) |
| Estonia | 2.43  (0.05) | 1952.3  (44.1) | 1.96  (0.06) | 635.4  (23.5) |
| Finland | 2.43  (0.04) | 914.0  (14.4) | 2.11  (0.05) | 376.6  (10.4) |
| France | 2.47  (0.13) | 755.0  (45.1) | 1.63  (0.17) | 205.2  (32.8) |
| Hungary | 4.33  (0.05) | 2711.1  (16.0) | 2.65  (0.04) | 925.5  (12.9) |
| Italy (Turin) | 1.97  (0.08) | 464.9  (32.1) | 1.08  (0.07) | 36.3  (24.6) |
| Lithuania | 2.96  (0.05) | 1715.2  (24.9) | 2.59  (0.08) | 524.7  (13.5) |
| Norway | 2.60  (0.05) | 883.1  (14.9) | 2.36  (0.06) | 497.3  (11.5) |
| Poland | 5.44  (0.06) | 1394.7  (6.3) | 2.78  (0.04) | 361.6  (4.3) |
| Scotland | 2.59  (0.22) | 1081.8  (102.5) | 1.87  (0.25) | 179.3  (18.8) |
| Slovenia | 2.71  (0.06) | 1074.5  (25.1) | 1.93  (0.07) | 360.3  (17.2) |
| Spain (Barcelona) | 2.13  (0.06) | 660.5  (24.6) | 1.55  (0.08) | 92.2  (21.1) |
| Spain (Basque Country) | 1.77  (0.05) | 450.1  (26.3) | 1.30  (0.08) | 84.4  (24.3) |
| Spain (Madrid) | 1.76  (0.05) | 542.7  (32.2) | 1.19  (0.07) | 588.2  (82.3) |
| Sweden | 2.05  (0.03) | 574.1  (9.9) | 2.03  (0.04) | 358.0  (7.3) |
| Switzerland | 2.44  (0.04) | 679.6  (11.2) | 1.62  (0.04) | 213.3  (8.8) |
| 1. Supply- and choice-oriented public systems | 2.75  (0.43) | 1057.5  (315.9) | 1.93  (0.14) | 368.6  (82.2) |
| 2. Performance- and primary care-oriented public systems | 2.36  (0.17) | 815.7  (123.0) | 2.26  (0.10) | 429.2  (42.9) |
| 3. Regulation-oriented public systems | 2.32  (0.15) | 937.0  (85.5) | 2.17  (0.04) | 571.2  (38.2) |
| 4. Low-supply and low performance mixed systems | 3.79  (0.65) | 1986.9  (387.1) | 2.60  (0.18) | 638.2  (147.7) |
