# Supplementary material for: Educational inequalities in mortality amenable to healthcare. A comparison of European healthcare systems
Source: PLoS One. 2020 Jul 2;15(7):e0234135. doi: 10.1371/journal.pone.0234135 (PMC7332057; doi:10.1371/journal.pone.0234135)
Supplement: Attachment — Submitted filename: response to reviewers.docx [file pone.0234135.s007.docx]

To both reviewers:

Thank you for valuable comments. See responses to specific points below.

Note that citations and references in the manuscripthas been updated in accordance with PlosOne’s preferences.

Note that a general language check also has been performed on the manuscript.

Reviewer 1

| **Reviewer 1 comment** | **Authors’ response** |
| --- | --- |
| Abstract 1.  The use of 'tertiles' is misleading as the three educational groups are not equally sized. Instead the authors should just say three groups. It would be helpful (in the body of the main manuscript) if the authors could confirm that they have taken the difference in size of the educational groups within each country into account in calculating the RII and SII values. | ‘Tertiles’ removed. In ‘Analyses’ section, we specify that the RII and SII accounts for the distribution of the population across educational groups. |
| Abstract 2.  The authors should mention the time period for which the mortality data pertain in the methods. | Done |
| Abstract 3.  It would be useful to provide some numerical results in the abstract, perhaps summarising the meta-analytical results. | Done |
| Abstract 4.  I will make further comments on the use of significance testing elsewhere, but I do not think the use of significant in the abstract is appropriate. | Use of ‘significant’ revised (and by and large removed) over entire manuscript. |
| Introduction 1.  In setting up the research question the authors use educational attainment as a pragmatic means of ranking the population to assess health inequalities. This should be made explicit that there is no attempt to use any theories of socioeconomic position or class to understand how educational attainment per se is related to the outcomes. This is okay for the purpose of this paper, but it would be helpful for this to be explicit. | Sentences on this is now added in the second paragraph. |
| Introduction 2.  The authors have misrepresented Julian Tudor Hart's seminal work on the inverse care law in the second paragraph. The full definition of this is, "The availability of good medical care tends to vary inversely with the need for it in the population served. This inverse care law operates more completely where medical care is most exposed to market forces, and less so where such exposure is reduced. The market distribution of medical care is a primitive and historically outdated social form, and any return to it would further exaggerate the maldistribution of medical resources." (https://www.thelancet.com/journals/lancet/article/PIIS0140-6736(71)92410-X/fulltext). This is important because the inverse relationship is explicitly linked to the degree of marketisation of the healthcare system - something that is only superficially addressed in the composite index used for the analysis in this paper. I did not follow the description and application provided here and would urge the authors to reconsider and redraft this section. | The inverse care law served a purpose in an earlier draft of this manuscript, but in its current state, we agree that it is misrepresented. Reference has been deleted and focus shifted away from the inverse care law. |
| Introduction 3.  I understand that the focus of the work here is in examining inequalities rather than mean outcomes. However, it would be helpful to acknowledge in paragraph three that the Nordic countries have comparably high life expectancy despite reasonably high inequality. | Highly relevant point, the end of this paragraph has been redrafted, referencing the high Nordic life expectancy. |
| Introduction 4.  (Relevant to the methods and discussion section too.) The definitions of amenable and avoidable mortality have been developed in order to distinguish the role and contribution of healthcare interventions. There is some discussion in the paper about these and the authors have taken the decision to change the accepted definitions in order to reduce the role of some causes (e.g. those closely related to substance use) as they view these are less amenable to healthcare intervention. In doing so the authors are implicitly acknowledging that none of the definitions of amenable or avoidable mortality are very accurate in that almost all causes of death are both socially/economically caused, and, to some degree, amenable to some healthcare intervention. It would be helpful to have a fuller discussion of what the use of the selected mortality codes do and do not show, and the limitations of these. It would be particularly helpful to discuss how this fits with the concepts of primary, secondary and tertiary prevention. | Fuller discussion of amenable mortality has been added. Emphasis has been added to how the classification in this manuscript rests on previous similar studies. |
| Introduction 5.  The discussion on the different means of classifying healthcare systems is very informative and interesting. It does however highlight the limitations of this way of classifying (similar to the limitations of the classifications of welfare state types). I am not an expert in the variety of healthcare systems across Europe but I did think that some of the groupings were very surprising. The link between restricting choice (which is an important means of preventing ineffective spending on low impact pharmaceuticals and specialist input) and funding is confounded - as the former limits the latter all other things being equal. The very small number of countries for which data were used in each category very much limits the extent to which it can be said that any differences in outcomes are due to the systems or other unmeasured confounding factors. Bringing together categories seems to further blunt any nuance within the typology and is another limitation that needs acknowledged. | Uncertainty due to small sample size is emphasized, ‘statistical test’ aspect has been de-emphasized.  Point on choice restriction-funding confounding is highly relevant, this has been added to the ‘Limitations’ section. Further limitations of the typology approach have been added. |
| Hypotheses 1.  Please edit the sentence with "...supply-healthcare systems Even through these systems...". | Done |
| Hypotheses 2.  I wasn't clear about the logic of the hypothesis that "high public involvement in the healthcare system and of high supply, free access and choice does not result in high educational inequality in amenable mortality". This runs counter to Tudor Hart's work about marketisation of healthcare. This may depend what is meant by 'public involvement', as the meaning of this is not clear to me. | We agree that the argument is unclear, ‘Hypothesis’ (Now ‘Expectations’ section has been redrafted. |
| Methods 1.  Please justify why the selection of countries was made. Were these the only countries for which data was available perhaps? | That is correct. This is now mentioned. |
| Methods 2.  The data used in the analysis is now quite old. This needs to be acknowledged and justified. | Age of data addressed in ‘Data’ section. Comparability issues related to old mortality data addressed in ‘Limitations’. |
| Methods 3.  The exclusion of 20% of Finns and non-Swiss nationals needs to be discussed further. To what degree do these create systematic biases? | Addressed in the ‘Limitations’ section. |
| Methods 4.  The correction factor used will not correct for systematic biases in non-linkage. This needs to be discussed as a limitation. | This is correct. Addressed in the ‘Limitations’ section. |
| Methods 5.  I wasn't convinced by the claims that ischaemic heart disease and heart failure are not amenable to healthcare intervention. Clearly it has both social and economic causes as well as being amenable to treatment. This again highlights the limitations of the amenable mortality measure and the attempt to dissociate the healthcare effects from the social and economic effects. | Added a more thorough discussion on amenable mortality as a concept, addressing among other the IHD issues. |
| Methods 6.  Could the authors justify why a pre-analysis protocol was not produced and published online? | This has to our experience not been a common practice in the social sciences. We considered the disadvantages (data dredging) of a pre-analysis protocol to be greater than the benefits (openness). |
| Methods 7.  I was unclear as to why the ISCED categories where grouped into three groups rather than used across all available data to service the regression modelling. Can this be justified? | The tripartite division is in line with many previous studies (cf. Mackenbach et al, 2017; Brekke et al., 2018). Further, this was the best available division which was comparable across all study countries. |
| Methods 8.  Omitting those without educational attainment recorded is problematic. For example, in some years in the UK this has been as high as 85% of the population and systematically different from the population mean (see https://www.ncbi.nlm.nih.gov/pubmed/25862252). | Valid point, thank You for reference. Addressed in the ‘Limitations’ section. |
| Methods 9.  It is described that mortality was "controlled for 5-year age groups". Do the authors mean that the data were age-standardised? If so, to what standard population? | Yes, to the European Standard Population. Reference added (Ahmad et al., 2001). |
| Methods 10.  The approach to the ANOVA analysis is well-described and appropriate. | ANOVA analyses output added to appendix. |
| Methods 11.  I found it somewhat implausible that cerebrovascular disease was classified as amenable but IHD was not. I worked as a GP at this time and if anything the opposite was true. | We rely on the previous classifications of the same data. We attempt to emphasize the ambiguity of the ‘amenable mortality’ concept. |
| Results 1.  The authors are over-reliant on statistical significance to make inferences (see https://www.bmj.com/content/322/7280/226.1). There is substantial confusion in the reporting about whether they are simply talking about the degree to which there could be random variation; small sample sizes and underpowering of the analysis; and the importance/policy relevance of the analysis. These are all conflated and strong conclusions are made about there being no differences in some cases when in fact this is simply likely to be due to small samples. | Descriptions of results and the following discussions and conclusions have been modified, reliance on statistical significance has been de-emphasized. Uncertainty due to small sample sizes has been addressed in ‘Analyses’ section (thank You for reference!) |
| Results 2  It would be best if the ANOVA results were simply put into a webappendix so that it can be readily available in the future and fully transparent. People move on and analyses get lost otherwise! | Done. |
| Results 3  It is important that the mortality rates for each ISCED group, and the proportion of the population in each ISCED group, and the mean for the total population, are provided in a table. | Educational distribution added to table A.1. |
| Results 4.  The sensitivity analyses should be shown in a webappendix. | Appendix tables updated. |
| Discussion 1.  In the first sentence the authors discuss equal access to healthcare. Do they mean equal or equitable? Being clearer about their definition of health inequality would also be helpful in this regard. | Sentence rewritten. |
| Discussion 2.  The use of Barcelona, Basque Country, Madrid and Turin is a major limitation and the authors should not make any generalisation to the countries overall in the use of these data. The points made about urban and rural inequalities are not relevant here. It is akin to generalising to the whole of the UK from London or Glasgow inequalities, either of which would be completely misleading. Within Spain the poorest regions are not included; the same is true for Italy. | Regional populations removed from typology estimates but kept in tables and figures as a reference. Meta- and ANOVA analyses are updated and changed accordingly. Point on urban/rural inequalities are removed from limitations section. |
| Discussion 3.  At the end of the first paragraph ("In line with more agency-based approaches...") the formulation seems to be that regulation is about preventing the rich using their resources to access healthcare. This seems a little inconsistent with the countries in the typology where there is a private healthcare sector which regulation does not limit access to. I'd urge the authors to redraft this section. | Made sense in an earlier draft. Section rewritten. |
| Discussion 4.  The second paragraph discusses some aspects of the inverse care law. There is a large primary care literature on this (see Graham Watt, Stewart Mercer, Mhairi Mackenzie and others for example) which could usefully be integrated into this section. | Useful reference. We have attempted to integrate the primary care perspective to the ‘Discussion’ section (though the Inverse Care Law has been taken out of the manuscript). |
| Discussion 5.  I think the robustness of the results of the paper are overplayed and the limitations under-recognised in the discussion. There are numerous issues with the measures of the exposure and outcome, as well as limited data availability and a high risk of confounding. Taken together it is quite difficult to be sure that the relationship described is robust. | This has been addressed in several sections, where reliance on statistical robustness has been de-emphasized. |
| Discussion 6.  A limitation not recognised in the use of a selection of mortality codes is competing causes. By removing IHD etc., people with multiple morbidity in middle age will be removed from the analysis and underestimate the mortality rates that would have occurred had the IHD not intervened. Comparing the results with all-cause mortality inequality would allow this to be discussed in more detail. | Comparison by sensitivity analyses with total all-cause mortality is added.  Point on competing causes has been addressed in first paragraph of ‘Discussion’ section. |
| Table 1  I was not clear what the numbers in the period column mean. Possibly months and years? | That is correct. Months was available only for some data sources. For clarity, months have therefore been deleted from the table. |
| Figure 1  It is not clear what the vertical dotted lines represent. | Dotted lines were not necessary and have been removed. |

Reviewer 2

| Reviewer comment | Authors’ response |
| --- | --- |
| The motivation of the study, focusing on specific elements of welfare regimes (in this case, health systems) rather than broader categories, is first discussed in the fourth paragraph. I think it would be helpful to lead with health systems, and then conceptualize their role in the broader discussion on health inequalities. | Healthcare systems has been addressed earlier in the introduction. |
| While the authors do a good job describing the typology of health systems, I believe one key aspect is missing: temporal information on the underlying variables. That is, the typology is based on measures that vary considerably over time, e.g., health expenditure per capita, number of GPs, public share of health expenditure, tobacco and alcohol consumption, etc. However, it is unclear which years were used to construct the original typology which raises at least two concerns: First, the typology might be based on recent data, which means that educational inequalities based on mortality data from ca. 2000-2005 (depending on the country, according to Table 1, p. 22) are related to characteristics of ‘current’ health systems. Second, if the typology is identified in years around the Millennium, i.e., similar to the health inequality measures, the implications for current health governance are different. In any case, I find the reader is unable to evaluate the analysis and conclusion due to this uncertainty. | Addressed in the third paragraph of ‘Limitations’ section. ‘Causal’ effect of health system type has been de-emphasized in accordance with several data limitations. |
| Data issues are another major concern for me. First, some of the data cover regional or urban populations. However, the hypotheses and typology of health systems are based on national characteristics (e.g., to what extent is the population of Madrid and/or its health system measures representative of Spain?, do these drive results?). Second, ischemic heart disease and heart failure were excluded due to their ‘strong association with life style factors such as smoking, alcohol consumption and obesity’ (p. 7). Yet for the typology of health systems, ‘healthcare performance was measured by indicators of tobacco and alcohol consumption …’ (p. 4). Thus, the performance measure does consider tobacco and alcohol, the mortality data does not. I think this inconsistency needs to be addressed. | Regional populations have been excluded from the typology estimates. Limitations have been discussed and clarity has (hopefully) been added with regards to the tobacco/alcohol issues in the 5^th^ paragraph of the ‘Limitations’ section. |
| The authors argue for the benefits of using health system typology as explanatory variable, while acknowledging several limitations (pp. 13-14). Particularly in view of the finding that health spending is important, I remain to be convinced why, under these circumstances, the typology is useful as an analytical category. In the concluding section, the authors ‘recommend future research on amenable mortality and morbidity to examine specific health policies’ (p. 16). Why does this study not already cover at least one of these aspects? | The sections on healthcare spending has been redrafted, since the empirical evidence supporting this claim was less robust. We have emphasized how this article uses a novel typology combined with individual-level mortality data as a starting point for discussing and exploring the mechanisms through which healthcare and health inequalities may be associated. |
| In the opening paragraph (p. 2), the authors mention numerous pathways through which education affects health outcomes and behavior. I would encourage them to use these insights and studies, and discuss the hypotheses (pp. 5-6) with a stronger focus on educational inequalities. | Helpful comment, this paragraph has been revisited in the ‘Discussion’ section. |
| In Figure 1, it would be helpful for the reader if it was indicated which clusters the countries belong to. A note should also explain how to interpret the estimates. | Clusters added in parentheses |
